# *Ante-mortem* and *Post-mortem* Inspection and Relationship between Findings in a North Albanian Pig Slaughterhouse

**DOI:** 10.3390/ani13061032

**Published:** 2023-03-12

**Authors:** Egon Andoni, Sonila Cocoli, Dino Miraglia, Claudia M. Balzaretti, Gabriele Brecchia, Bizena Bijo, Laura Menchetti, Laura Musa, Giulio Curone, Stella Agradi, Ilirian Kumbe, Pellumb Zalla, Edison Gjoni, Xhilola Bixheku, Marta Castrica

**Affiliations:** 1 Faculty of Veterinary Medicine, Agricultural University of Albania, 1029 Kamez, Albania; 2 Department of Veterinary Medicine, University of Perugia, Via San Costanzo 4, 06126 Perugia, Italy; 3 Department of Veterinary Medicine and Animal Sciences, University of Milan, Via dell’Università 6, 26900 Lodi, Italy; 4 School of Biosciences and Veterinary Medicine, University of Camerino, Via Gentile III da Varano, 62032 Camerino, Italy; 5 National Authority of Veterinary and Plant Protection, Rr “Jordan Misja”, Pall 14/1 shk, 1001 Tirane, Albania; 6 Quality Assurance Agency in Higher Education, Rruga Durrsit, Nr 219, 1001 Tirane, Albania

**Keywords:** meat inspection, pig slaughter, carcass and offal condemnation

## Abstract

**Simple Summary:**

In European Union abattoirs, the safety of meat is dependent on the favorable opinion from an official veterinarian, in accordance with the current legislation. From this perspective, the feedback generated from the *ante-mortem* visit and the *post-mortem* inspection can be investigated to control the health and welfare conditions of the animals in the pre-slaughter phases. From this perspective, we evaluated the *ante-mortem* and *post-mortem* inspection outcomes of slaughtered pigs in northern Albania and correlated the results in order to gain insight into the conditions and injuries of pigs slaughtered outside the European context and to extend knowledge on the possible relationship between *ante-mortem* and *post-mortem* relief. Dyspnea and tail, skin, and ear lesions were the most frequently observed conditions before slaughter, while pleuritis, pneumonia, liver alterations, white spots on the liver, and pericarditis were the most frequent lesions after slaughter. A significant increase in the total number of *post-mortem* findings was also observed as the number of *ante-mortem* findings increased. Overall, the prevalence of the findings observed in this study falls within the broad range of the data in the literature, but additional information should be collected during meat inspection so as to better understand the relationship between *ante*- and *post-mortem* outcomes.

**Abstract:**

In June 2014, Albania was granted EU candidate status, thus starting a process of compliance with the membership criteria. In this context, a modern meat inspection approach in line with the European legislation was applied to a pig slaughterhouse in northern Albania in order to investigate the *ante-mortem* (AM) and *post-mortem* (PM) conditions and the relationship between these findings. For this purpose, 3930 pigs divided into 35 batches were evaluated over a 3-month period. The most frequent AM conditions recorded were tail lesions and dyspnea (9.1%), followed by skin (8.9%) and ear lesions (8.5%), while in the PM inspections, pleuritis was the most frequently observed condition (10.2%), followed by pneumonia (8.5%), liver alterations (5.7%), milk spot liver (3.8%), and pericarditis (3.3%). With the exception of liver alterations, the other PM lesions mentioned were positively associated with lesions on the ears (OR = 1.036; *p* < 0.001) and skin (OR = 1.026; *p* = 0.011) and dyspnea (OR = 1.021; *p* = 0.005), confirming the link between these variables and the health and welfare conditions of pigs on farms. Overall, the evidence that emerged from this Albanian slaughterhouse can be considered in line with other European contexts, especially in light of the considerable variability in the data present in the literature.

## 1. Introduction

Meat inspection (MI) performed by official veterinarians (OVs), with the support of trained official assistants where necessary, involves various tasks, including an (i) examination of the animals before slaughter, the *ante-mortem* inspection (AMI); (ii) inspection of the meat immediately after slaughter, the *post-mortem* inspection (PMI); and (iii) further checks, e.g., regarding animal identification, animal health and welfare aspects, and food hygiene [1]. MI’s general aim is to detect animal diseases or health problems at an early stage in order to prevent the occurrence and spread of foodborne diseases and to preserve the quality of foods of animal origin in order to protect public health [2]. In addition, official control of the abattoir provides a wealth of information that can be used in epidemiological studies and for the development and implementation of appropriate management programs on the farm level [3,4,5,6]. In the European Union, the modalities for conducting MI activities and official controls, in general, are regulated by Regulation (EU) No. 2019/627 and legislated by Regulation (EU) No. 2017/625 of the European Parliament and Council. It is also important to note that between 2011 and 2013, the EFSA published six scientific opinions on public hazards linked to MI and recommended possible improvements or alternative methods for the above-mentioned activities [7]. In particular, palpation or incisions should be omitted during PMI in the case of “normal” animals, as the benefit of this approach is disproportionate to the risks involved (the risk of cross-contamination is higher). Therefore, in the case of domestic swine, since 2014, Regulation (EU) No. 2014/219 has required only a visual inspection of low-risk animals. However, where the epidemiological data on the animals’ condition, the food chain data or AMI, and/or *post-mortem* visual inspections indicate relevant abnormalities with possible risks to public health, animal health, or animal welfare, the carcasses and offal of pigs must undergo additional *post-mortem* procedures, including incision and palpation [2,8,9].

In this context, Albania started the process of gaining access through the robust European legislative scheme in April 2009 [10], and in June 2014, candidate status was given to Albania. The process of access involves different negotiations proposed by the EU so as to reach the health and hygiene standards defined by the EU acquis in order to grant full access, including the transposition of and harmonization with the European Regulations concerning official controls, food safety, animal welfare, and public health, taking into account the following main aspects: (i) the transmission of zoonosis and (ii) foodborne diseases.

In Europe, the overall production of pig meat was around 332.7 million tonnes in 2021, and among the EU-27, the countries with the highest production of pig meat in 2021 were Spain, with 58.4 million tonnes, Germany (51.9 million tonnes), and France (23.3 million tonnes) [11]. Regarding Albania, in 2021, the production of pig meat was 189.2 thousand tonnes [11], and the production was mostly located in the north of the country, where the regions of Lezha and Shkodra produced 34.6% and 25.4% of the total production respectively [12]. In these areas, both intensive indoor closed systems and free-range small farm models are observed. 

In this context, although Albania represents a small segment of pig production compared to the production capacities of the various European countries, there is a need for legislative and practical adaptation to the European model with regard to (i) free trade and (ii) Albania’s future possibility of joining the EU. For these reasons, the aim of this study was to evaluate, for the first time, the sanitary conditions of pigs slaughtered in northern Albania (near Shkodra) through a retrospective observational study conducted through an approach in line with the current European legislation based on the scheme of Ghidini et al. [13], with minor adaptations. In addition, an investigation of the relationship between the conditions or lesions found during AMI and PMI was carried out in order to identify predictive significance.

## 2. Materials and Methods

### 2.1. Data Collection

During a 3-month period (February–April), retrospective observational research was conducted on a total of 3930 animals divided into 35 batches (a batch corresponded to all the pigs slaughtered in one day) in a small-scale pig abattoir (approximately 900 animals slaughtered per week) located near Shkodra in northern Albania.

The slaughterhouse accepts both pigs imported from EU countries (Greece, Hungary, and Slovakia) and swine from local pig farms located around the abattoir. The animals are between 4 and 5 months, with a total live body weight around 80 to 100 kg, reared using both traditional and free-range methods. The pigs are always slaughtered within 8 h of unloading at the slaughterhouse. AMI and PMI were performed by trained official veterinarians with at least 3 years of experience. A detailed form based on *ante-mortem* and *postmortem* relief, as proposed by Ghidini et al. [13] with minor adaptations, was used to record all the specific conditions and lesions observed in each animal and in each carcass (Table 1). Pre-slaughter aspects (e.g., farm origin, type of farming, etc.) were not investigated in this study.

### 2.2. Statistical Analyses

Descriptive statistics were first used to present the data as absolute and relative frequencies with the mean prevalence and 95% confidence intervals [14]. The associations between the number of PM conditions and observed AM lesions were investigated by Poisson regression, including the batch size as an offset variable [1]. Univariable models were built, where each AM was included as an independent variable and each PM was a dependent variable. The focus was on the total AM lesions and AM conditions with a prevalence >5% (i.e., skin, ear, and tail lesions, and dyspnea), while the examined PM conditions were those with a mean prevalence higher than 3% (i.e., pneumonia, pleuritis, pericarditis, milk white spot, and liver alterations). The results were expressed as odds ratios (OR) with 95% confidence intervals (CI) and the *p*-value based on Wald statistics. The data were analyzed using IBM SPSS Statistics, version 25, and the level of statistical significance was set as *p* ≤ 0.05.

## 3. Results

### 3.1. Descriptive Results

**Figure 1:**
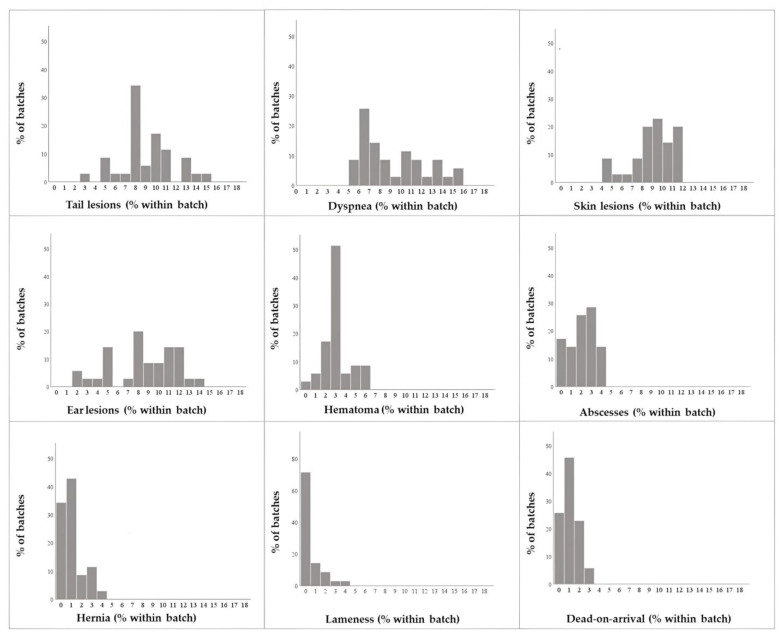
Frequency distributions of within-batch prevalence of *ante-mortem* conditions/lesions and within-batch mortality during transport recorded among slaughtered pigs (*N* = 35 batches).

During the 3-month period, a total of 35 batches representing 3930 animals were examined (mean ± standard deviation = 112 ± 20 animals/batch; range = 80–160 animals/batch). The most prevalent AM lesions recorded among the batches were tail lesions (mean = 9.1%; 95%CI = 8.1–10.0%) and dyspnea (mean = 9.1%; 95%CI = 8.1–10.1%), followed by skin (mean = 8.9%; 95%CI = 8.1–9.6%) and ear lesions (mean = 8.5%; 95%CI = 7.4–9.6%). There were no batches free of these lesions. The relief of hematomas was observed with an average frequency of 3.1% (95%CI = 2.6–3.6%), while the lowest prevalence was found for lameness (mean = 1.1%; 95%CI = 0.8–1.4%) and hernia (mean = 1.1%; 95%CI = 0.7–1.4%) (Figure 1 and Table S1 and Table 2). Concerning deaths on arrival, a mean rate of 0.5% (95%CI = 0.2–0.8%) was recorded based on 10 batches, while 0.15% of the subjects died in the pens.

**Figure 2:**
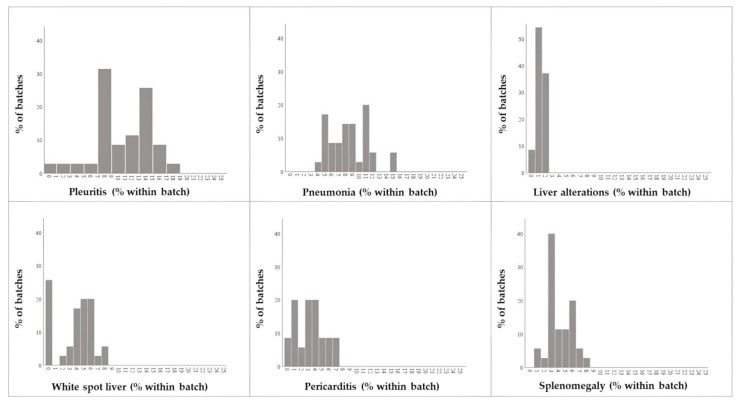
Frequency distributions of the highest within-batch prevalence of *post-mortem* lesions recorded in slaughtered pigs (*N* = 35 batches).

The most frequently observed PM conditions were pleuritis (mean = 10.2%; 95%CI = 8.8–11.6%), pneumonia (mean = 8.5%; 95%CI = 7.6–9.5%), and liver alterations (mean = 5.7%; 95%CI = 4.9–6.4%). Conversely, peritonitis and splenomegaly were only found in three batches. Figure 2 and Table S2 and Table 3 show the full details. Finally, the whole-carcass condemnation was 0.66%.

### 3.2. Relationship between Ante- and Post-mortem Lesions

**Figure 3:**
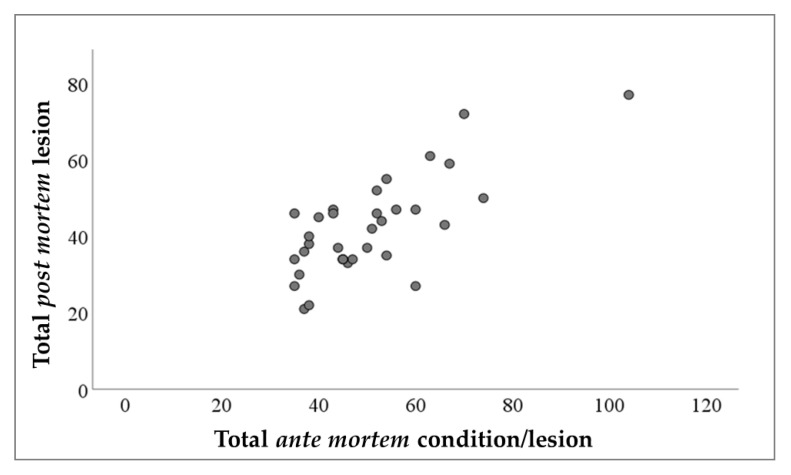
Scatter plot showing the association between the total *ante-mortem* and total *post-mortem* relief (OR = 1.007, 95%CI = 1.004–1.010; *p* < 0.001). The model included the batch size as an offset variable.

A positive association was found between the total count of PM lesions and the total number of AM finds (OR = 1.007, 95%CI = 1.004–1.010; *p* < 0.001; Figure 3). The most influential AM conditions in regard to the total PM outcomes were ear lesions (OR = 1.036, 95%CI = 1.023–1.050; *p* < 0.001), dyspnea (OR = 1.021, 95%CI = 1.006–1.037; *p* = 0.005), and skin lesions (OR = 1.026, 95%CI = 1.006–1.047; *p* = 0.011). Specifically, ear lesions increased the odds of pleuritis (*p* ≤ 0.001), pericarditis (*p* = 0.013), and milk spot liver (*p* = 0.002), while AM dyspnea increased the odds of pneumonia (*p* = 0.001) and pericarditis (*p* = 0.031; Table 4). Finally, skin lesions increased the odds of pleuritis (*p* = 0.038) and milk spot liver (*p* = 0.004).

## 4. Discussion

*Ante-mortem* and *post-mortem* meat inspection is an important tool used to monitor and ensure food safety and consumer health and to study the prevalence and incidence of animal diseases [15]. In this regard, abattoir inspections can help to enhance pig welfare standards by providing feedback to the farmers, who can then take appropriate interventions to improve the management and prevention of certain animal diseases on the farm level, thus reducing losses through lower rates of carcass condemnation, trimming, and downgrading [16].

### 4.1. Ante-mortem Findings

Dyspnea is described as a clinical sign characterized by open-mouth breathing and increments in oxygen demand. It can occur in various respiratory diseases, but noninfectious stressogenic causes such as temperature, environmental factors, or management can also cause rapid and wheezy breathing. In the current study, dyspnea was the most frequently observed *ante-mortem* condition, along with tail biting (9.1%) (Table 2). This finding was not unexpected, considering that the outcomes of pleuritis (10.2%) and pneumonia (8.5%) were reported most frequently during the PM inspection (Table 3). Conversely, in the study conducted by Ghidini et al. [1], only 0.01% of pigs destined for slaughter presented with dyspnea, even though the most frequently observed *post-mortem* lesions concerned the respiratory system. In pigs with pneumo-lesions, the demand for oxygen and excess carbon dioxide may be accentuated when stress increases [17]. Therefore, in our research, the welfare conditions during the transport of the animals to the slaughterhouse may have been a determining variable in triggering the dyspnea symptoms [17].

Tail biting is an unpredictable and abnormal behavior which can be observed on farms, during the transport of the animals to the abattoir, or in pens before the slaughter process, where overcrowded conditions and poor animal welfare occur [16]. Pigs that perform tail biting are also likely to perform other forms of damaging behavior, such as ear biting [18]. Tail lesions during abattoir inspection have already been proposed, together with skin lesions, as potential iceberg indicators of pig health and welfare [19]. Lesions due to tail biting elevate stress levels and have a negative impact on farm economy due to reduced weight gain [20]. The prevalence of serious tail biting outbreaks, including fresh signs of tissue damage, varies widely between studies, with a prevalence of between 2 and 12% for undocked pigs [21]. The percentage of tail lesions in our research was 9.1%, which was the most common finding during the *ante-mortem* inspection. In the study conducted by Maisano et al. [22], which included around 10,000 pigs, tail biting was never observed, while Ghidini et al. [1] recorded a very low prevalence (<1%), but many animals were tailless. On the contrary, Valros et al. [20] found that about 50% of the pigs examined had a tail that was not fully intact. However, the same authors stated that tails with signs of bites or bruises but without fresh lesions should not be counted. Accordingly, when comparing the rates of prevalence of tail biting lesions in the existing literature, it is important to consider the great variations in the sampling methods and definitions of tail biting cases [20,21]. Currently, there are many dissimilarities in the classification system of these lesions, which should instead be harmonized, not only to render the feedback to farmers more detailed and specific but also to obtain more reliable epidemiological data. For this reason, much information on the prevalence of tail biting in the EU may not be correct [20].

Skin lesions can originate from several pathologies already present on the farm during breeding or derive from trauma during the loading/unloading or the transport of animals. They are also classified as important indicators of animal health and welfare, because they can be detected on carcasses up to 11 weeks after their onset [23]. Nevertheless, the majority of the skin lesions observed in slaughterhouse result from traumatic conditions due to fighting when groups of different animals are transported together to the slaughterhouse [24]. The prevalence of skin lesions observed in this research was 8.9%, almost equal to that described by Ghidini et al. [1] (9.1%). However, it should be emphasized that in the current work, 4–5-month-old pigs with a weight of 90–100 kg were evaluated, whereas in the study conducted by Ghidini et al. [1], the pigs were 9 months old and weighed 170 kg. The increased weight at slaughter and the attainment of sexual maturity in females are, in fact, considered as risk factors for skin lesions, and they also increase the aggressiveness of individuals as a result of the progressive decrease in space [24]. Additional information should be collected on the characteristics of these lesions (e.g., acute or chronic, from trauma or bite) at the abattoir in order to better understand the origin of the problem. 

Ear lesions are a growing problem on pig farms, with a recent study reporting a 100% farm-level prevalence on 31 farms, with a median of 6.97% of animals affected [19]. In our study, ear lesions represented the fourth most frequently observed condition in AMI, with a mean prevalence of 8.5%. These findings are close to the data recorded by Bottacini et al. [24], who reported a prevalence of 9.0%. In the study conducted by Ghidini et al. [1], ear lesions were the third most frequently observed condition during the AM inspection, albeit with a lower mean prevalence (3.3%). Ear lesions represent a common finding in swine, reflecting damage behavior mainly occurring in weaners. Breeding conditions, long transport distances, the loading density, and associations between separate farms or different age group ranges of the pigs are the main factors influencing these records [19,25,26,27,28,29,30,31], but we were unable to precisely determine the events that led to the onset of the injuries. Unfortunately, like other indicators of animal welfare and health, the simple detection of ear lesions at the slaughterhouse without further information on the type of injury and when it occurred limits the effectiveness of these outcomes as retrospective tools. We also agree with other authors (Bottacini et al., 2018) about the need for additional information to enable targeted investigations and specific corrective actions on farm.

Regarding animals identified as dead-on-arrival, according to internationally recognized animal welfare experts, the transport mortality rate should remain below the 0.1% threshold [32]. In the current study, dead-on-arrival pigs were present in ten of the thirty-five batches, with a mean prevalence well above the indicated limit (0.5%), without considering the animals that subsequently died (0.15%). A better situation was observed by Ghidini et al. [1], who reported an arrival death rate of 0.04%, and by Guardone et al. [25] in their 10-year retrospective survey (0.09%). However, in the latter study, the authors found considerable variability over the years, with the prevalence peaking at 0.26%. Pigs are very susceptible to stress, and transport is considered a critical phase in pig meat chains in which several unfavorable conditions can occur simultaneously. Long distances, high stocking densities, the mixing of different animals, and excessively warm temperatures are some of the factors that may contribute to higher stress levels and, consequently, deaths during transport and on arrival [26,27,28,29]. The prevalence found in the current study, therefore, highlights the need to review and improve transport conditions. However, the absence of detailed information on this phase does not allow for a proper analysis of the problem, which we consider necessary in order to take targeted corrective actions [33].

### 4.2. Post-mortem Findings

Respiratory diseases are among the most problematic conditions affecting indoor intensive systems in conventional swine production [34]. Pleuritis signifies an inflammation of the pleura that can appear in acute or chronic forms. Chronic forms are characterized by the presence of abscesses, fibrosis, and adherence of the lungs to the chest wall [35]. Pleurisy is a routine finding in slaughterhouses, resulting in significant losses for the pig industry due to a reduced carcass weight, poor feed efficiency, and increased number of days to slaughter [36]. In most cases, pleuritis follows the inflammation of other organs of the thorax, mostly the lungs, but the pericardium may also be affected [37]. Pneumonia is defined as inflammation of the lungs, which is commonly found in pigs, causing death at all ages. Lesions due to enzootic pneumonia, characterized by cranio-ventral consolidation and caused by viruses and numerous bacteria, particularly Mycoplasma hyopneumoniae and *Pasteurella multocida*, as well as pleurisy, whose main causative agent is *A. pleuropneumoniae*, are reported as the most frequent findings at pig slaughterhouses [38]. Although this condemnation category includes several conditions, such as pleuropneumonia, catarrhal bronchopneumonia, or pleuritis, the latter has been referred to as the main cause of condemnation [39]. Pleuritis and pneumonia represented the most common findings in this research, observed in 10.2% and 8.5% of all cases. In studies conducted on pigs slaughtered in Italy, Maisano et al. [22] reported a prevalence of 25.8% for pleuritis and 17.1% for pneumonia, while Ghidini et al. [1,13] reported 17.2% for pleuritis, 8.2% and 6.4% for pneumonia, and 15.5% for pleuropneumonias. The rates of the prevalence of pneumonias and pleuropneumonias (3.5% and 6.9% respectively) reported by Guardone et al. [25] in a 10-year retrospective study were lower, while a rate of only 0.1% of polyserositis cases was found by Ceccarelli et al. [40] in a 7-year survey. These findings indicate a certain variability in the reported anatomopathological findings and, in particular, in the frequency of their observation, in light of which the prevalence of respiratory diseases observed in the current study does not seem to be particularly worrying. However, when comparing data, a certain degree of dissimilarity in the evaluation and recording of lesions by the competent authorities must be taken into account, particularly for pneumonic diseases in pigs [41]. 

Liver alterations are often observed in *post-mortem* findings and cause partial or total condemnation of the organ. In most cases, these lesions are related to milk spots, but they may also be a consequence of hepatitis, perihepatitis, necrosis, and cirrhosis [13,25,40]. In this study, liver alterations affected 100% of the batches, with an average prevalence of 5.7%. A similar rate of prevalence was found by Ghidini et al. [1] (4.9%), whereas in the study conducted by Guardone et al. [25], liver alterations affected only 2.6% of pigs, accounting for 6.9% of liver condemnations. In the retrospective study of Ceccarelli et al. [40], the liver was the organ most frequently condemned, but only 3.9% of the cases were not milk spot liver. In the current study, however, liver alterations other than white spot resulted in more than half of all liver seizures (59%). Certainly, it is the latter data that deviate most from the literature, and therefore, further investigation on lesions is required.

Milk spot liver is an accepted terminology used to describe the white foci of fibrosis present in the liver parenchyma resulting from an inflammatory reaction caused by the migration of Ascaris suum. [42]. Milk spots tend to appear asymptomatically, while liver lesions are observed in slaughterhouses during *post-mortem* inspection. The presence of milk spots represents the main cause of liver exclusion by official veterinarians [25,40], resulting in significant economic losses due to offal condemnation [43]. The prevalence of milk spot liver in the present study was 3.8%, close to the value observed by Kongsted et al. [44] for conventional indoor herds (4.6%) but significantly lower than the same authors’ findings for conventional and organic free-range farms (12.2% and 15.8% respectively). In these farming systems, outdoor access and straw bedding are predisposing factors for Ascaris suum infection [45]. Similarly, on intensive farms, the excessive animal density facilitates the spread of the parasite within groups (Thomsen et al., 2001). Prevalence rates higher than ours were also reported by Ghidini et al. [1] (7.6%), as well as Maisano et al. [22] and Scollo et al. [46] (25% and 24%, respectively). In these cases, however, all the farms raised heavy pigs aged between 9 and 10 months for the production of Protected Designation of Origin (PDO) products, whereas in our study, the pigs were 4–5 months old at the time of slaughter. This early age may have contributed to the low prevalence, although other variables that were unknown to us, such as the parasite control plans adopted on the farms, should be considered [46]. The main cause of cardiac condemnations is pericarditis, a non-specific pathological finding resulting from infections, which can be viral or fungal but are mainly bacterial, particularly Haemophilus parasuis, *Pasteurella* spp., Mycoplasma spp., and Streptococcus spp. [47]. The prevalence of pericarditis (3.3%) detected in this study was rather low, overlapping with the values recorded by Ghidini et al. [13] (3.2%) and Maisano et al. [22] (4.3%) in Italian abattoirs and by Correia-Gomes et al. [47] (3.5%) in Northern Ireland. Other recent research, also conducted in Italy, reported higher observation rates, such as 7.9%, reported by Ghidini et al. [1] and 6.9%, reported by Guardone et al. [25]. In the study of Ceccarelli et al. [40], the heart was the second most frequently removed organ (10.8%) due to pericarditis (99.8% of cases). Therefore, from an epidemiological point of view, we consider that the observation rate found in the present work is not of particular concern, even considering the threshold of attention proposed by the Welfare Quality protocol for pigs [48], defined as 5% prevalence of pericarditis at slaughter. 

### 4.3. Relationship between Ante- and Post-mortem Lesions

Currently, there is a lack of information in the literature regarding the relationship between *ante-mortem* conditions of pigs and *post-mortem* outcomes [1]. Identifying possible correlations could render the risk-based approach more reliable and simplify the tasks of the competent authorities during *post-mortem* inspection. In addition, retrospective information for assessing the health and welfare of animals on farms could be improved. In this study, there was a significant increase in the total number of PM findings as the AM findings increased (*p* < 0.001), with ear lesions, skin lesions, and dyspnea being the most significant variables. In fact, all the PM lesions that had a frequency of observation (prevalence) greater than 3% (Table S2) were positively associated with at least one of the three *ante-mortem* conditions mentioned above (Table 4), with the exception of liver alterations. In particular, ear lesions were the most influential condition on the total PM lesions (OR = 1.036; *p* < 0.001), increasing the odds of pericarditis (*p* = 0.013), pleuritis (*p* < 0.001), and milk spot liver (*p* = 0.002) by 5.8%, 5.3%, and 6.1%, respectively (Table 4). The latter two PM findings were also recorded more frequently in animals with skin lesions, with increased probabilities of 4.2% for pleuritis (*p* < 0.038) and 10.1% for milk spot liver (*p* < 0.004). The associations between the pre-existing pathological conditions and skin and ear lesions of slaughtered pigs indirectly confirm the link between these variables and the health and welfare conditions of pigs on farms [49]. In fact, damage to the skin and ears, together with lameness and tail biting lesions, are included by the EFSA in the ‘good health injury’ category [22,49]. In this regard, several on-farm risk factors that affect the occurrence of these findings in slaughtered pigs have been recognized. For example, the increase in the herd and pig density has been identified as a predisposing condition for skin, ear, and tail injuries, as well as parasitoses, including ascaridiosis [50], pleuritis, and pneumonia [51]. At the same time, similar problems have been reported on free-range farms, where outdoor access to animals increases the risk of respiratory diseases [52], joint and body injuries [34,52,53], and ascaridiosis [45]. However, even though tail biting lesions were the most frequently reported AM condition in our study, no *post-mortem* findings on the animals were positively associated with outcomes of this abnormal behavior. It would therefore appear that pigs with tail bites are different from those with ear and skin lesions, and that the causes of these outcomes are not the same. However, the conditions and management of the herds of origin of the animals, as well as the transport phase, were not investigated in this study; thus, further considerations regarding this issue are not possible. In the study conducted by Ghidini et al. [1], correlations between AM and PM findings also emerged, but they partly differed from ours. For example, ear lesions and lameness increased the OR of abscess observation by 32% and 42%, respectively. Skin lesions, on the other hand, as in our study, were positively associated with milk spot liver (OR: +24%) but not with pleuritis. Regarding the AM symptoms of dyspnea, in our study, these were associated with increases of 5.6% and 4.9% in the observation rates of pericarditis and pneumonia, respectively, whereas in the investigation performed by Ghidini et al. [1], this relationship was not found. In particular, in the *ante-mortem* inspection, the authors observed symptoms of dyspnea in only 0.01% of the batches, compared to 7.82% for pericarditis and 8.16% for pneumonia reported in the PM inspection. Thus, it is evident that the correlation between AM and PM variables does not necessarily imply a causal relationship, although they may have a common origin. 

## 5. Conclusions

From an epidemiological point of view, the rates of prevalence of lesions detected in this study are not dissimilar to those described in other European contexts. On the other hand, the considerable variability in the data in the literature adds to the difficulties of comparison. In general, the frequency of observations of *ante*- and *post-mortem* findings is linked to the actual clinical and anatomopathological conditions of animals transported to the slaughterhouse but can also be influenced by the accuracy with which the competent authority reports these conditions. In addition, for non-European countries such as Albania, the different legislative framework is another variable that must be considered when examining the data. However, it is important to emphasize that the findings of this study are limited to a single abattoir and a few farms and do not necessarily capture the real condition in Albania.

With regard to the relationships between certain anatomopathological findings and skin and ear lesions found in the current study, we should emphasize the importance of these conditions as indicators of the health and welfare of pigs on the farm. At the same time, however, equally important AM indicators, such as tail biting, did not increase the frequency of observation of any PM lesions, being irrelevant in this aspect. In this regard, the associations reported by other authors, which partly disagree with ours, generate further questions about causal relationships that need to be investigated further. Thus, in order to offer a correct interpretation of AM/PM correlation relationships, further studies are required in order to fill the gaps in the information on this issue that is currently available in the literature. It will also be necessary to set up a longitudinal experimental approach, in which the variables considered are observed in the same animals, on the farm, after transport to the slaughterhouse, and in the *ante*- and *post-mortem* inspections.

## Figures and Tables

**Table 1:** Form adopted for *ante-mortem* (AM) and *post-mortem* (PM) relief, partially adapted from Ghidini et al. [13].

Condition/Lesion	Description	AM/PM *
Dyspnea	Wheezing breathing.	AM
Skin lesions	Bruises or injuries on several parts of the body.	AM
Tail lesions	Fresh blood, inflammation, infection, or missing tail tissue.	AM
Ear lesions	Bruises, wounds, missing ear parts, or ear necrosis.	AM
Lameness	Severely lame pigs.	AM
Hematoma	Blood in various parts of the body.	AM/PM
Erysipelas	Diamond-shaped skin lesions.	AM
Abscess	All abscesses, excluding lung and liver abscesses.	AM/PM
Hernia	Umbilical or inguinal hernia.	AM/PM
Cachexia	Poor body condition score.	AM
Anemia	Pale mucosae.	AM
Dead-on-arrival	Dead pigs on arrival.	AM
Dead in box	Dead pigs in pens.	AM
Pneumonia	Pneumonia and the outcomes of pneumonia, including lung abscesses.	PM
Pleuritis	Inflammation of the pleura with the presence of adherence or fibrine.	PM
Pericarditis	Inflammation of the pericardium with the presence of fibrine.	PM
Jaundice	Generalized jaundice.	PM
Splenomegaly	More than 50% of the organs affected.	PM
Arthritis	Inflammation of one or more joints.	PM
Peritonitis	Inflammation of the peritoneum.	PM
White spot liver	Milk spot lesions in the cortex of the liver.	PM
Insufficient bleeding	Presence of blood in the muscle.	PM
Liver alterations	Hepatitis and outcomes of hepatitis, steatosis, and liver necrosis.	PM
Enteritis	Intestine thickening with or without hemorrhages or necrosis.	PM
Generalized lymphadenitis	Increased volume of lymph nodes.	PM

* AM: *ante-mortem*, PM: *post-mortem*.

**Table 2:** Number of positive batches and within-batch prevalence of recorded *ante-mortem* conditions/lesions and within-batch mortality during transport of slaughtered pigs (*N* = 35 batches).

Condition/Lesion	No. of Positive Batches	% of Positive Batches	Within-Batch Prevalence (%)		
			Mean	95% CI *	Range (Min-Max)
Tail lesions	35	100.0	9.1	8.1–10.0	3.0–15.0
Dyspnea	35	100.0	9.1	8.1–10.1	5.5–15.0
Skin lesions	35	100.0	8.9	8.1–9.6	4.0–11.7
Ear lesions	35	100.0	8.5	7.4–9.6	2.0–14.0
Hematoma	34	97.1	3.1	2.6–3.6	0.0–6.0
Abscess	25	71.4	1.2	0.9–1.6	0.0–4.0
Erysipelas	9	25.7	0.7	0.3–1.2	0.0–4.0
Anemia	24	68.6	1.1	0.8–1.4	0.0–3.0
Cachexia	22	62.9	0.8	0.5–1.1	0.0–3.3
Lameness	26	74.3	1.1	0.8–1.4	0.0–3.0
Hernia	23	65.7	1.1	0.7–1.4	0.0–4.0
Dead-on-arrival	10	28.6	0.5	0.2–0.8	0.0–4.0
Dead in box	6	17.1	0.1	0.0–0.2	0.0–0.9

* Confidence interval.

**Table 3:** Number of positive batches and within-batch prevalence of recorded *post-mortem* lesions in slaughtered pigs (*N* = 35 batches).

Lesion	No. of Positive Batches	% of Positive Batches	Within-Batch Prevalence (%)		
			Mean	95% CI *	Range (Min-Max)
Pleuritis	34	97.1	10.2	8.8–11.6	0.0–17.0
Pneumonia	35	100.0	8.5	7.6–9.5	4.0–15.0
Liver alterations	35	100.0	5.7	4.9–6.4	1.0–8.0
White spot liver	26	74.3	3.8	2.9–4.7	0.0–8.0
Pericarditis	32	91.4	3.3	2.5–4.0	0.0–7.0
Hematoma	34	97.1	3.1	2.6–3.6	0.0–6.0
Splenomegaly	3	8.6	1.3	1.1–1.5	0.0–2.0
Hernia	23	65.7	1.1	0.7–1.4	0.0–4.0
Abscess	11	31.4	0.7	0.3–1.1	0.0–4.0
Arthritis	17	48.6	0.6	0.4–0.8	0.0–2.0
Jaundice	12	34.3	0.4	0.2–0.7	0.0–2.0
Enteritis	9	25.7	0.3	0.1–0.5	0.0–2.0
Insufficient bleeding	6	17.1	0.2	0.0–0.3	0.0–1.0
Peritonitis	3	8.6	0.1	0.0–0.2	0.0–1.0
Generalized lymphadenitis	0	-	-	-	-

* Confidence interval.

**Table 4:** Relationship between the counts of *post-mortem* and *ante-mortem* relief observed in slaughtered pig batches (*N* = 35). Poisson regressions were used to analyze the data. Univariable models were built, where each PM was included as a dependent variable and each AM was an independent variable. Only AM conditions with a prevalence >5% and PM with a mean prevalence >3% were examined. For all the models, batch size was included as an offset variable.

PM * Lesion	AM * Condition/Lesion	OR **	95% Wald Confidence Interval for OR **		*p*-Value
			Lower	Upper	
Pneumonia	Dyspnea	1.049	1.019	1.081	0.001
	Skin lesions	1.010	0.969	1.053	0.637
	Tail lesion	1.019	0.996	1.042	0.104
	Ear lesions	1.019	0.993	1.047	0.159
Pleuritis	Dyspnea	1.021	0.992	1.051	0.157
	Skin lesions	1.042	1.002	1.084	0.038
	Tail lesions	1.002	0.980	1.024	0.869
	Ear lesions	1.051	1.026	1.077	<0.001
Pericarditis	Dyspnea	1.056	1.005	1.109	0.031
	Skin lesions	1.011	0.943	1.084	0.755
	Tail lesions	1.012	0.974	1.052	0.551
	Ear lesions	1.058	1.012	1.106	0.013
Splenomegaly	Dyspnea	1.008	0.962	1.056	0.752
	Skin lesions	1.013	0.953	1.077	0.681
	Tail lesions	0.988	0.954	1.024	0.514
	Ear lesions	1.033	0.993	1.075	0.104
White spot liver	Dyspnea	0.993	0.946	1.043	0.784
	Skin lesions	1.101	1.031	1.174	0.004
	Tail lesions	1.026	0.992	1.061	0.136
	Ear lesions	1.063	1.022	1.106	0.002
Liver alterations	Dyspnea	0.968	0.928	1.010	0.134
	Skin lesions	0.985	0.936	1.037	0.576
	Tail lesions	0.963	0.933	0.995	0.022
	Ear lesions	0.988	0.955	1.023	0.504

* AM: *ante-mortem*, PM: *post-mortem*. ** Odds ratio.

## Data Availability

The data presented in this study are available on request from the corresponding author.

## Supplementary Materials

The following supporting information can be downloaded at: https://www.mdpi.com/article/10.3390/ani13061032/s1, Table S1: Prevalence of recorded *ante-mortem* conditions and/or lesions in slaughtered pigs (*N* = 3930 pigs), Table S2: Prevalence of recorded *post-mortem* lesions in slaughtered pigs (*N* = 3930 pigs).
