# Supplementary material for: Ante-mortem and Post-mortem Inspection and Relationship between Findings in a North Albanian Pig Slaughterhouse
Source: Animals (Basel). 2023 Mar 12;13(6):1032. doi: 10.3390/ani13061032 (PMC10044242; doi:10.3390/ani13061032)
Supplement: Supplementary file 1 [file animals-13-01032-s001.zip › animals-2197110-supplementary.pdf]

Table S1. Prevalence of recorded *ante mortem* conditions and/or lesions in slaughtered pigs (N=3,930 pigs).

Condition/lesion	No. of pigs	Prevalence
**Tail lesions**	353	8.98
**Dyspnea**	352	8.96
**Skin lesions**	344	8.75
**Ear lesions**	329	8.37
**Hematoma**	119	3.03
**Abscess**	51	1.30
**Anemia**	41	1.04
**Hernia**	39	0.99
**Lameness**	38	0.97
**Cachexia**	31	0.79
**Erysipelas**	28	0.71
**Dead-on-arrival**	22	0.56
**Dead in box**	6	0.15

Table S2. Prevalence of recorded *post mortem* lesions in slaughtered pigs (N = 3,930 pigs).

Lesion	No. of pigs	Prevalence
**Pleuritis**	388	9.87
**Pneumonia**	336	8.55
**Liver alterations**	213	5.42
**White spot liver**	148	3.77
**Pericarditis**	119	3.03
**Hematoma**	119	3.03
**Splenomegaly**	46	1.17
**Hernia**	39	0.99
**Abscess**	28	0.71
**Arthritis**	21	0.53
**Jaundice**	16	0.41
**Enteritis**	10	0.25
**Insufficient bleeding**	6	0.15
**Peritonitis**	3	0.08
**Generalized lymphadenitis**	0	-
